# Supplementary material for: Digital measurement of ocular microtremor in Parkinson’s disease: Protocol for a pilot study to assess reliability and clinical validation
Source: PLoS One. 2025 Jan 8;20(1):e0313452. doi: 10.1371/journal.pone.0313452 (PMC11709268; doi:10.1371/journal.pone.0313452)
Supplement: Attachment — Submitted filename: Reponse to Reviewers.docx [file pone.0313452.s002.docx]

Reviewer Response: PLOS One

***Digital measurement of ocular microtremor in Parkinson’s Disease: Protocol to assess reliability and clinical validation.***

The authors would like to thank both reviewers for taking their time to perform a thorough review of our work. Please see our responses to your comments below.

| **Reviewer comment** | **Author Response** |
| --- | --- |
| 1. Please ensure that your manuscript meets PLOS ONE's style requirements, including those for file naming. The PLOS ONE style templates can be found at  <https://journals.plos.org/plosone/s/file?id=wjVg/PLOSOne_formatting_sample_main_body.pdf> and  <https://journals.plos.org/plosone/s/file?id=ba62/PLOSOne_formatting_sample_title_authors_affiliations.pdf> | Thank you, the manuscript has been edited as appropriate. |
| 2. Thank you for stating the following financial disclosure:  “This study is based at the Physiotherapy Innovation Laboratory (Website: [www.pi-lab.co.uk](http://www.pi-lab.co.uk), Twitter: @Physio_In_Lab) and has been funded by a Northumbria University PhD studentship in collaboration with Head Diagnostics Ltd. (Dublin, Ireland). Dr Samuel Stuart is supported, in part, by a Parkinson’s Foundation Post-doctoral Fellowship for Basic  Scientists (PF-FBS-1898-18-21) and a Clinical Research Award (PF-CRA-2073). Private Physiotherapy Educational Foundation funding also supported this research (PPEF – #368; PI: Stuart).”  Please state what role the funders took in the study.  If the funders had no role, please state: "The funders had no role in study design, data collection and analysis, decision to publish, or preparation of the manuscript."  If this statement is not correct you must amend it as needed.  Please include this amended Role of Funder statement in your cover letter; we will change the online submission form on your behalf. | Thank you. The funders had no role in study design, data collection and analysis, decision to publish, or preparation of the manuscript. This has been added to the cover letter. |
| 3. Thank you for stating the following in the Acknowledgments Section of your manuscript:  “This study is based at the Physiotherapy Innovation Laboratory (Website: [www.pi-lab.co.uk](http://www.pi-lab.co.uk), Twitter: @Physio_In_Lab) and has been funded by a Northumbria University PhD studentship in collaboration with Head Diagnostics Ltd. (Dublin, Ireland). Dr Samuel Stuart is supported, in part, by a Parkinson’s Foundation Post-doctoral Fellowship for Basic  Scientists (PF-FBS-1898-18-21) and a Clinical Research Award (PF-CRA-2073). Private Physiotherapy Educational Foundation funding also supported this research (PPEF – #368; PI: Stuart).”  We note that you have provided funding information that is currently declared in your Funding Statement. However, funding information should not appear in the Acknowledgments section or other areas of your manuscript. We will only publish funding information present in the Funding Statement section of the online submission form.  Please remove any funding-related text from the manuscript and let us know how you would like to update your Funding Statement. Currently, your Funding Statement reads as follows:  “This study is based at the Physiotherapy Innovation Laboratory (Website: [www.pi-lab.co.uk](http://www.pi-lab.co.uk), Twitter: @Physio_In_Lab) and has been funded by a Northumbria University PhD studentship in collaboration with Head Diagnostics Ltd. (Dublin, Ireland). Dr Samuel Stuart is supported, in part, by a Parkinson’s Foundation Post-doctoral Fellowship for Basic  Scientists (PF-FBS-1898-18-21) and a Clinical Research Award (PF-CRA-2073). Private Physiotherapy Educational Foundation funding also supported this research (PPEF – #368; PI: Stuart).”  Please include your amended statements within your cover letter; we will change the online submission form on your behalf. | Funding information has been removed from the manuscript. |
| 4. When completing the data availability statement of the submission form, you indicated that you will make your data available on acceptance. We strongly recommend all authors decide on a data sharing plan before acceptance, as the process can be lengthy and hold up publication timelines. Please note that, though access restrictions are acceptable now, your entire data will need to be made freely accessible if your manuscript is accepted for publication. This policy applies to all data except where public deposition would breach compliance with the protocol approved by your research ethics board. If you are unable to adhere to our open data policy, please kindly revise your statement to explain your reasoning and we will seek the editor's input on an exemption. Please be assured that, once you have provided your new statement, the assessment of your exemption will not hold up the peer review process | The authors wish to revise this statement based on the fact this is a study protocol and therefore are no data related to the manuscript. |
| 5. Please review your reference list to ensure that it is complete and correct. If you have cited papers that have been retracted, please include the rationale for doing so in the manuscript text, or remove these references and replace them with relevant current references. Any changes to the reference list should be mentioned in the rebuttal letter that accompanies your revised manuscript. If you need to cite a retracted article, indicate the article’s retracted status in the References list and also include a citation and full reference for the retraction notice. | Thank you. References have been revised and Vancouver stye has been applied. |

**REVIEWER 1**

| **Reviewer comment** | **Author Response** |
| --- | --- |
| Reviewer #1: This is a very well conceptualized and constructed protocol. All necessary components are included and adequately detailed. This protocol is IRB approved and has been registered on clinicaltrials.gov. I have no reservations in recommending this protocol for publication at this time. | Thank you for taking the time to consider our manuscript and for your helpful comment. |

**REVIEWER 2**

| **Reviewer comment** | **Author Response** |
| --- | --- |
| Reviewer #2: • Is the protocol technically sound and planned in a manner that will lead to a meaningful outcome and allow testing of the stated hypotheses?  Yes, with the following caveats:  The protocol indicates that patients will be recruited when they first present at clinic with suspected PD.  Those that are confirmed as PD go into the PD group, those that get some other diagnosis (or are healthy) go into the ‘not PD’ group.  Superficially this makes good sense, at it allows the researchers to compare two groups at the point of diagnosis.  However, the non-PD group will be extremely heterogeneous and may include people with no illness at all, people with non-PD motor disorders and people with other illnesses that could compromise their eye-movements such as stroke or Alzheimer’s disease. I think this could be a potential problem for interpretation, especially if the study finds no group differences. | Thank you for this comment.  This is the first time that ocular-microtremor has been measured with a hand-held and non-invasive device in a clinic setting with any clinical population. Therefore, we aimed to explore the OMT measurements of anyone attending the PD clinic, which includes both those who may and those that may not have received a PD diagnosis.  We agree that the non-PD group (i.e., those without a definitive clinical diagnosis of PD) may be a heterogeneous group, but we cannot be certain of this due to this being the first time that OMT has been measured in this way. Additionally, interpretation will relate to the OMT measure range for those with definitive PD, not just the pure frequency score, which may aid differentiation between groups. The results will allow us to make inferences about what the OMT score range may be for someone with PD in a clinic setting, as well as view if this is different or overlapping from those without definitive clinical PD diagnosis (who may have a larger range of OMT frequency scores as a group). This initial exploratory work will aid the development of future studies that may be able to target specific non-PD groups, e.g., for differential diagnosis. |
| The justification for the sample size is not very convincing. The purpose of the power analysis is establish the sample necessary to observe a statistically significant difference between the two groups. Given there is prior evidence that OMT differs in PD and controls I would expect to see a formal power analysis to support the sample of 30, irrespective of whether or not the authors wish to draw conclusions about the clinical value of the test. | This is the first time that a novel hand-held digital, non-invasive device has been used to measure OMT in PD. Only one previous study on OMT in PD exists, involving a sample of just 22 PD (Bolger, 1999). However, this previous study that measured OMT in PD used a different invasive and non-portable technology, which is completely different from the present study. Therefore, there are no previous studies to base a specific sample size on for this study.  Additionally, this is an exploratory pilot study, and therefore a sample size calculation is not necessary(Julious, 2005; In, 2017). The purpose of a pilot study is to gather data for future trials to calculate more specific sample size estimates from. To ensure we would have enough data and subjects to perform relevant analysis we selected a sample size of 30 per group for the main aims, which is greater than the previous PD study (which found group differences; therefore, we expect to see similar differences).  References:  Julious, S. A. (2005). Sample size of 12 per group rule of thumb for a pilot study. *Pharmaceutical Statistics: The Journal of Applied Statistics in the Pharmaceutical Industry*, *4*(4), 287-291.  In J. (2017). Introduction of a pilot study. *Korean journal of anesthesiology*, *70*(6), 601–605. https://doi.org/10.4097/kjae.2017.70.6.601  Bolger, C., Bojanic, S., Sheahan, N. F., Coakley, D., & Malone, J. F. (1999). Ocular microtremor in patients with idiopathic Parkinson’s disease. *Journal of Neurology, Neurosurgery & Psychiatry*, *66*(4), 528-531. |
| Why is only the PD group getting a 2nd assessment in the home? It seems necessary to test reliability in both groups in the home to make a valid conclusion about the reliability of iTremor, especially as people homes are much more variable than the lab, so it would not be very surprising to see at least some variation in scores when testing moves from controlled lab environments to homes. | Thank you for your comment. Validation is specific to the population being tested, and this is particularly true for clinical populations. In this case it is vital to perform reliability and clinical validation in PD specifically, as they are the clinical group of focus within this study. We are interested only in the analytical (reliability) and clinical (known groups, convergent etc.) validity of the OMT measures in PD.  The iTremor device has already been tested for validity (including reliability) in health adults in several previous studies (Kenny, 2013a, Kenny, 2013b, Kenny, 2013c). Further reliability testing in healthy controls would not inform the reliability of the OMT measures in PD, which is the focus of this study. The healthy control group is included in the study for an aspect of clinical validation, specifically known groups validity, as we would expect there to be a difference in OMT between these groups.  If future studies are interested in the reliability of OMT in healthy older adults, then it will be possible to do so.  ***References*:**  Kenny, E., Coakley, D., & Boyle, G. (2013a). Ocular microtremor measurement using laser-speckle metrology. *Journal of biomedical optics*, *18*(1), 016010-016010.  Kenny, E., Coakley, D., & Boyle, G. (2013b). Biospeckle in the human sclera and impact on laser speckle correlation measurement of eye tremor. *Journal of Biomedical Optics*, *18*(9), 097009-097009.  Kenny, E., Coakley, D., & Boyle, G. (2013c). Ocular microtremor measurement using laser-speckle metrology. *Journal of biomedical optics*, *18*(1), 016010-016010. |
| How will usability and acceptability be assessed? The authors mention reporting descriptions of usability and acceptability but not how this date will be collected (e.g. is it based on the researchers reflection? Will patients be asked? will metrics like the time it takes to perform the test be measured?) | Thank you for your comment. The terminology has been modified in the statistical analysis section to align with the study aims, specifically the term ‘acceptability’ has been removed from the analytical validation aim II text, as usability and acceptance are not included in the aims of the study. Aim II specifically focusses on the feasibility of OMT measurement in the clinic setting, and several feasibility outcomes have been listed to be descriptively reported (e.g., number of adverse events). |
| **Minor comments from reviewer:** | |
| Introduction Line 81-83 : sentence doesn’t make sense. I think because the authors don’t explain what ability is being referred to in line 81. | This has been edited for clarity.  *“For example, pro-saccade performance (directing gaze towards a target) has been identified in predicting decline in PD, highlighting the potential benefits of accurate eye tracking in detecting these [2].”* |
| Line 84: the authors argue that that main problem for using eye-movements to diagnose PD is that some patients don’t understand the instructions on some tasks. They allude to the anti-saccade task, but are not explicit.  I didn’t quite follow this line of argument, as earlier in the para it is argued that prosaccades are in fact the most best for predicting decline.  I would also be surprised if non-dementing PD patients had problems understanding anti-saccade or memory guided saccade tasks (at least, no more so than similarly aged controls).  The issue of medication is more problematic, but of course if a patient is already on medication isn’t it likely they have been diagnosed with PD, so the use of eye-movement data for diagnosis is no longer necessary? (whereas it it might still be useful for progression monitoring) | Thank you for this comment.  For clarity, the anti-saccade task involves inhibiting the natural response by directing the gaze away (in the opposite direction) of the target. The pro-saccadic task would involve directing the gaze towards the target. These tasks are useful in examining the voluntary and flexible control of eye movements. This has been added to the manuscript as follows: *“In other research, again using a 12 hour ‘OFF’ period, similar effects were reported showing that levodopa slowed reflexive pro-saccades (directing gaze towards a target), but the authors also reported improved voluntary anti-saccades (directing gaze in the opposite direction of target)(12).”*  Whilst PwPD may not be demented, mild cognitive impairment is very common amongst PwPD even at the time of diagnosis (Aarsland., 2011, Weil., 2018). This could impact performance in the anti-saccade task. What we are getting at is the need for more passive rather than active monitoring of eye movement deficits. OMT could provide a useful biomarker of disease severity, irrespective of cognitive impairment/ task understanding. We have edited the text accordingly to enhance clarity.  *References:*  Aarsland, D., Brønnick, K., & Fladby, T. (2011). Mild cognitive impairment in Parkinson’s disease. Current neurology and neuroscience reports, 11, 371-378.  Weil, R. S., Costantini, A. A., & Schrag, A. E. (2018). Mild cognitive impairment in Parkinson’s disease—what is it?. Current neurology and neuroscience reports, 18, 1-11.  In regard to medication, participants on anti-Parkinson’s medication will have a diagnosis of PD. These participants can still be used to identify group differentiation between PwPD and HC. The avenue for testing PwPD ON and OFF medication was to explore whether changes in OMT frequency exist. This is not yet known. Previous studies tested PwPD whilst ON medication. |
| Line 304-306: the text suggests patients will be told to abstain from taking their meds, then a week later the team will visit. I assume this not the plan, so this phrasing needs to be tightened up | Step 3b, for the purpose of reliability assessment, participants were asked to take their morning dose of anti-Parkinson’s medication at the same time as they had in step 3a.  This has been clarified in the manuscript:  *“Participants from step 3a will be invited to participate in an additional home based assessment to explore the reliability of OMT measurement in PD using the iTremor ONE device. For this, we then visit the participant at the same time as step 3a, one week later. To perform the test re-test reliability, we ask participants to take their medication at the same time as they took it for step 3a.”* |
